# A Study on the Mechanisms and Performance of a Polyvinyl Alcohol-Based Nanogenerator Based on the Triboelectric Effect

**DOI:** 10.3390/ma17184514

**Published:** 2024-09-14

**Authors:** Wuliang Sun, Junhui Dong, Xiaobo Gao, Baodong Chen, Ding Nan

**Affiliations:** 1 School of Materials Science and Engineering, Inner Mongolia University of Technology, Hohhot 010051, China; sunwuliang@jx-nano.cn; 2 College of Chemistry and Chemical Engineering, Inner Mongolia University, Hohhot 010021, China; 3 Beijing Institute of Nanoenergy and Nanosystems, Chinese Academy of Sciences, Beijing 101400, China; gaoxiaobo@binn.cas.cn

**Keywords:** polyvinyl alcohol, triboelectric nanogenerator, contact electrification

## Abstract

Polyvinyl alcohol (PVA), a versatile polymer, is extensively used across many industries, such as chemicals, food, healthcare, textiles, and packaging. However, research on applying PVA to triboelectric nanogenerators (TENGs) remains limited. Consequently, we chose PVA as the primary material to explore its contact electrification mechanisms at the molecular level, alongside materials like Polyethylene (PE), Polyvinylidene fluoride (PVDF), and Polytetrafluoroethylene (PTFE). Our findings show that PVA has the highest band gap, with the smallest band gap occurring between the HOMO of PVA and the LUMO of PTFE. During molecular contact, electron transfer primarily occurs in the outermost layers of the molecules, influenced by the functional groups of the polymers. The presence of fluorine atoms enhances the electron transfer between PVA and PTFE to maximum levels. Experimental validation confirmed that PVA and PTFE contact yields the highest triboelectric performance: *V*_OC_ of 128 V, *I*_SC_ of 2.83 µA, *Q*_SC_ of 82 nC, and an output power of 384 µW. Moreover, P-TENG, made of PVA and PTFE, was successfully applied in self-powered smart devices and monitored human respiration and bodily movements effectively. These findings offer valuable insights into using PVA in triboelectric nanogenerator technologies.

## 1. Introduction

Energy is a fundamental pillar in the advancement of human society. As science and technology progress, the scarcity of conventional energy sources escalates, worsening environmental contamination. In this era, dominated by the Internet of Things and sensor networks, the urgent demand for energy has highlighted TENGs as a key emerging energy conversion technology [1]. TENGs utilize the triboelectric effect, generating static electricity through friction to convert, often untapped, mechanical energy into electrical energy. This sustainable, clean, and self-powered method provides a promising solution for powering electronic devices [2,3]. Characterized by high output and energy conversion efficiency at low frequencies, TENG technology offers numerous advantages, including diverse material options, simple fabrication, low cost, with no need for external power, as well as being eco-friendly and sustainable. These benefits make TENGs a promising technology for applications in wearable electronics, flexible devices, self-powered sensors, and large-scale blue energy harvesting, driving sustainable energy development and utilization [4,5,6]. To date, the field of triboelectric charging has experienced rapid growth, covering aspects such as architectural design, material enhancement, performance optimization, power management, and exploratory applications [2,7,8,9]. Tribology, a complex process, involves contact or sliding between two materials. Various localized physical and chemical reactions occur during this interaction, such as deformation, heat generation, and surface layer formation [10]. The foundational, scientific phenomenon of TENG technology is contact electrification, wherein charge generation arises from the contact and separation of objects. Mechanical friction aids, but is not essential to, charge transfer. Almost all material types, including polymers, semiconductors, inorganic materials, and metals, can charge through contact electrification [11,12].

Polymeric materials, with their unique functional groups, mechanical properties, and physicochemical characteristics, are widely used in TENG technology [13,14]. In TENGs, electron-withdrawing groups include fluorine (-F), ester (-COOR), nitrile (-CN), carboxyl (-COOH), acyl (-CON), and nitro (-NO_2_); electron-donating groups comprise amine (-NH_2_), hydroxyl (-OH), amide (-CONH), and alkoxy (-OR) [15,16]. These functional groups enhance electron transfer and capture during the frictional contact–separation process. Moreover, polymeric films, notable for their flexibility, stretchability, processability, and lightweight nature, have been positioned as core materials in modern TENG technology.

Polyvinyl alcohol (PVA), a semicrystalline polymer, is typically found in powdered form and synthesized through a two-step process. Synthesis begins with the free-radical polymerization of vinyl acetate into poly (vinyl acetate), which is then hydrolyzed to produce PVA [17]. Incomplete hydrolysis allows for PVA with varying degrees of hydrolysis, resulting in different solubility profiles and molecular weights. Consequently, PVA features a backbone of C-C macromolecular chains and numerous hydroxyl functional groups [18]. As a versatile molding material, PVA has been shaped into various forms, including films and coatings with high tensile strength, flexibility, and excellent odor barrier properties [19,20]. PVA is highly regarded as a triboelectric film due to its excellent hydrophilicity, good film-forming ability, mechanical flexibility, biocompatibility, chemical stability, tunable surface properties, and effective dielectric properties, making it ideal for efficient and sustainable energy harvesting applications. However, research on the role of PVA in triboelectric nanogenerators remains in the early stages [21,22,23].

In this study, we selected polyvinyl alcohol (PVA) as the primary material to systematically analyze its contact electrification mechanisms in response to various materials, its impact on the performance of a TENG, and certain practical applications. Initially, using Density Functional Theory (DFT), we investigated the contact electrification mechanisms between PVA and the representative polymers (PE, PVDF, and PTFE). The analysis covered the electrostatic potential, energy gap, and intermolecular charge transfer. The results show that PVA has a higher HOMO–LUMO gap, beneficial for electron transfer. When PVA contacts other polymers, electron transfer primarily occurs in the outermost electron layers, significantly influenced by the functional groups. The experimental results indicate that contact between PVA and PTFE yielded the highest electrical output. Applying these materials in a triboelectric nanogenerator (P-TENG) demonstrated their potential to power smart devices and monitor human respiration and bodily movements. This research lays a critical foundation for the application of PVA in triboelectric nanogenerators (TENGs).

## 2. Materials and Methods

### 2.1. DFT Method

Within this study, DFT calculations were conducted on the electrostatic potential, the energy levels of the LUMO, and the HOMO of all the investigated polymers, using the Gaussian 16 software suite. Moreover, charge transfer studies of the polymers were performed by employing the hised method within the DMol3 module of the Materials Studio 2016 software package.

### 2.2. Preparation of the P-TENG

PVA, with a molecular weight (*M*_n_) of 80,000 and a concentration of 12% *w*/*v* in distilled water, was dissolved in deionized water and the solution was prepared by stirring at room temperature until fully dissolved. The prepared PVA solution was then loaded into a 20 mL syringe, mounted on a syringe pump. Electrospinning was carried out with a voltage of 20 kV, a 0.5 mL/h flow rate, and a collection distance of 16 cm, to fabricate a fibrous membrane. The resulting nanofiber membrane was subjected to a drying process. The dried PVA fiber membrane (thickness 95 μm) was cut into 4 cm × 4 cm and a copper electrode was attached to the backside. Similarly, PE (thickness 40 μm), PVDF (thickness 50 μm), and PTFE (thickness 50 μm) were cut to the same size and copper electrodes were affixed to their backsides. Finally, the assembly of the friction nanogenerator (P-TENG), comprising dual electrodes, was completed.

### 2.3. Scanning Electron Microscope (SEM)

Our PVA film underwent a sputter coating with gold, during the experimental procedure. Subsequently, we utilized a scanning electron microscope (SU8020, Hitachi High-Tech Corporation in Tokyo, Japan) to observe the surface morphology of the nanofibrous membrane. To determine the diameter of the fibers, we employed the image processing software ImageJ (Image-Pro Plus 6) and randomly selected 50 fibers for measurement.

### 2.4. Atomic Force Microscope (AFM)

A comprehensive investigation was conducted on the surface topology and interlayer height distribution of diverse membranes using a Multimode 8 AFM, produced by Bruker Corporation. The AFM instrument boasts a maximum scanning area of 90 μm × 90 μm, with a spatial resolution that can attain 10–20 nm. This advanced precision surface analysis technique provides an accurate and quantitative delineation of the microstructures on the sample surface.

### 2.5. Electrical Performance Measurements

The open-circuit voltage, short-circuit current, and transferred charge of the P-TENG were measured using a programmable electrometer (Keithley Instruments model 6514). A linear motor (Linmot E1100) was employed to actuate the P-TENG under various parameters.

## 3. Results

**Figure 1:**
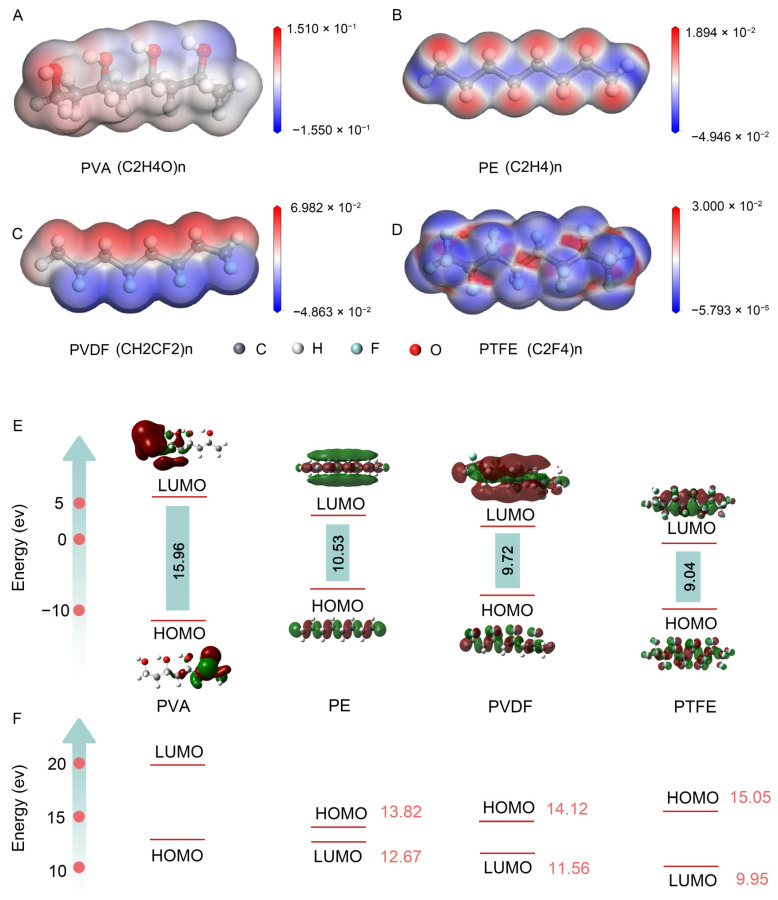
Electron density isosurface diagrams of PVA (**A**), PE (**B**), PVDF (**C**), and PTFE (**D**). (**E**) Simulated frontier orbital energy levels and calculated HOMO–LUMO energy gaps for PVA, PE, PVDF, and PTFE. (**F**) Orbital energy difference diagrams for PVA, PE, PVDF, and PTFE.

Molecular electrostatic potential plays a crucial role in explaining electrophilic and nucleophilic reactions and in identifying hydrogen bonding sites [24]. Figure 1 illustrates the electrostatic potential maps of various polymers, with red indicating positive potential (electron deficient) areas, and blue indicating negative potential (electron rich) areas. In Figure 1A, the O-H bonds in PVA are in a positive potential area, indicating lower electron density. In Figure 1B, the C-H bonds in PE are in the positive potential region, indicating that they are more likely to lose electrons. In Figure 1C, the C-H bonds in PVDF are in the positive potential region, while the C-F bonds are in the negative potential region. Lastly, in PTFE (Figure 1D), the C-F bond is in a negative potential region, showing a higher electron-accepting ability compared to other polymers. Overall, the positive potential of PVA is more likely to react with the negative potential of polymers such as PE, PVDF, and PTFE. This electrostatic potential mapping analysis provides a solid foundation for predicting and understanding the charge transfer phenomena between different polymers.

In molecular characteristic studies, the lowest unoccupied molecular orbital (LUMO) and the highest occupied molecular orbital (HOMO) are crucial for analyzing the charge transfer capabilities in contact electrification (CE). Figure 1E shows the distribution of the LUMO and the HOMO in PVA. The LUMO is primarily located on the left side of the molecule, while the HOMO clusters are on the right, possibly due to the asymmetric distribution of hydroxyl (-OH) groups. The data in Figure 1 show that the LUMO energies for PVA, PE, PVDF, and PTFE are 5.78 eV, 2.49 eV, 1.38 eV, and 0.23 eV, respectively, with corresponding HOMO energies of 10.18 eV, 8.04 eV, 8.34 eV, and 9.26 eV. Although these polymers share similar carbon chain skeletons, PVA has the largest HOMO–LUMO gap (15.96 eV), influenced by its specific functional groups.

This study conducts detailed calculations of the HOMO–LUMO gaps between PVA and other polymers to assess their potential for inter-polymer electronic transitions. Figure 1F shows the LUMO–HOMO gaps between PVA and the polymers PE, PVDF, and PTFE, measured at 13.82 eV, 12.67 eV, and 11.56 eV, respectively. Among these, PVA and PTFE have the smallest HOMO–LUMO gaps, suggesting that in potential electron transfer reactions, PVA is likely to donate electrons from its HOMO, while PTFE, along with PVDF and PE, tends to accept electrons into their LUMOs. Based on the band gap data from this study, the hierarchy of the electron-accepting capacity in charge transfer reactions with PVA is PTFE (HOMO–LUMO 12.67 eV), followed by PVDF (HOMO–LUMO 11.56 eV), and PE (HOMO–LUMO 9.95 eV). Compared to other polymers, these findings indicate that PTFE has the potential for a more significant electron transfer process.

Our study used DFT simulations to investigate the charge transfer (CT) between different polymer molecules upon contact. The validity of this method is supported by other experimental research. The CT effects relate to the interactions among the interfacial atoms of the materials, as confirmed in DFT-based research on both metal–polymer and liquid–polymer systems [25,26,27,28]. For this calculation, we selected PVA as the reference material and examined PE, PVDF, and PTFE to assess the charge transfer of PVA during the CE process. To ensure comparable results, we used a consistent number of polymer molecular chains, allowing the side functional groups to dominate the CE simulation. Consequently, we expect the CE simulation outcomes to be primarily influenced by the functional groups’ electron-donating and accepting properties.

**Figure 2:**
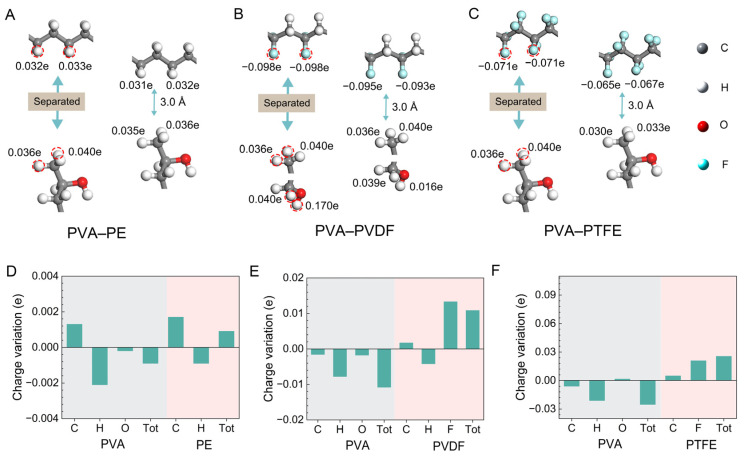
(**A**) Atomic charge transfer at the PVA–PE interface. (**B**) Atomic charge transfer at the PVA–PVDF interface. (**C**) Atomic charge transfer at the PVA–PTFE interface. (**D**) Charge transfer of different atoms in PVA–PE. (**E**) Charge transfer of different atoms in PVA–PVDF. (**F**) Charge transfer of different atoms in PVA–PTFE. Note that the columns represent the total transferred charge of the atoms in the polymers. (Note: The atoms marked by the red dashed circles match the charges given in the figure).

Building on prior research (Figure 1), we aligned the HOMO of PVA with the LUMO orbitals of PE, PVDF, and PTFE, to calculate the charge transfer conditions, as shown in Figure 2. In the PVA–PE system, we found that in an equilibrium state, with the total energy minimized to about 3 Å, there was an increase in the charge of the hydrogen atoms in PE at the interface and a decrease at the interface of PVA (Figure 2A), indicating charge migration. Additionally, we analyzed the specific charge transfer characteristics of individual atoms in the system. After contact with PVA (Figure 2D), the charge structure of PE was rearranged to achieve new stability. This reconfiguration resulted in a slight charge loss in the hydrogen atoms in PVA, while some hydrogen atoms in PE transferred electrons to carbon atoms, increasing their charge. Similar charge redistributions occurred in the PVA–PVDF and PVA–PTFE systems (Figure 2B,C, respectively). In the PVA–PVDF system (Figure 2E), fluorine atoms gained extra charge due to their high electron affinity, with hydrogen atoms in PVDF also transferring charge to them. In the PVA–PTFE system (Figure 2F), the hydrogen atoms in PVA lost charge, while the fluorine atoms in PTFE gained charge, following the electron structure reorganization at the interface. Furthermore, the electronic structure and charge distribution within polymers will change due to interfacial electronics regulation.

**Figure 3:**
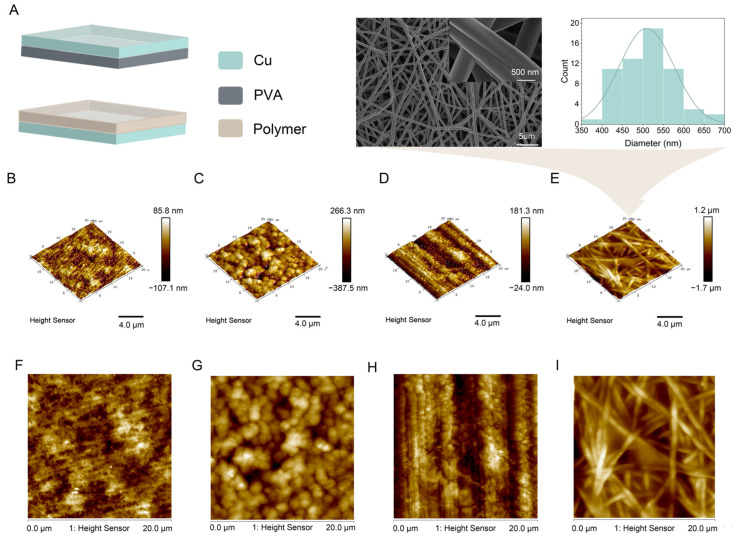
(**A**) Structure of the P-TENG. (**B**) Three-dimensional topography of PE film. (**C**) Three-dimensional topography of PVDF film. (**D**) Three-dimensional topography of PTFE film. (**E**) Three-dimensional topography of PVA film, corresponding electron microscopy images, and fiber diameters. (**F**) Phase image of PE film. (**G**) Phase image of PVDF film. (**H**) Phase image of PTFE film. (**I**) Phase image of PVA film.

To assess the impact of different membranes on the electrical characteristics of PVA, we conducted triboelectric experiments featuring the contact–separation friction between PVA against PE, PVDF, and PTFE. Copper electrodes were attached to both sides to construct a vertical contact–separation P-TENG, as depicted in Figure 3A. Atomic force microscopy (AFM) was used to obtain phase and topographical maps of these materials, with the results shown in Figure 3B–I. The analysis revealed distinct structural morphologies for each membrane. The PE surface (Figure 3B,F) displayed a compact, evenly dispersed, array of polymeric clusters, leading to a smoother surface topology. Conversely, PVDF (Figure 3C,G) featured irregular hill-like structures, contributing to increased film roughness. The additional fluorine atoms in PTFE (Figure 3D,H) enhanced the molecular chain stability, resulting in regular aggregates. Overall, all the fabricated membranes showed relatively smooth surface topologies. Surface roughness significantly influences the efficiency of triboelectric nanogenerators (TENGs) by altering the contact dynamics between triboelectric materials. Rougher surfaces increase the effective contact area, enhancing the frictional interaction and, thereby, boosting the charge transfer during the triboelectric effect. This leads to higher charge density and improves the electrical output. To enhance the charge transfer capabilities, PVA was electrospun into nanofiber membranes, characterized as smooth, homogenous, and coherent, with an average diameter of about 509 nm (Figure 3E,I).

**Figure 4:**
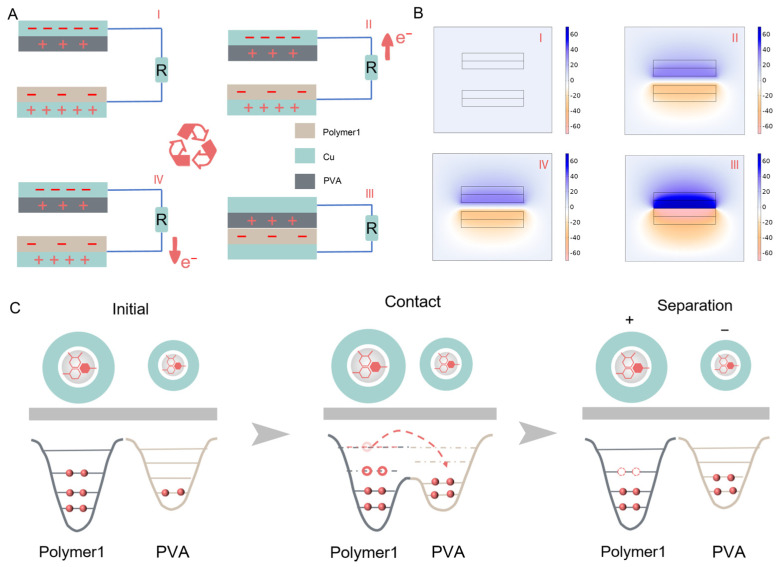
(**A**) Schematic diagram of the working principle of the P-TENG. (**B**) Contact–separation process of P-TENGs and corresponding electric potential distribution. (**C**) Schematic diagram of an atomic-level electron cloud/potential well model describing the contact electrification process of P-TENGs.

The working mechanism of the P-TENG, illustrated in Figure 4A, consists of two types of polymer materials and two corresponding electrode layers connected to a load circuit. When the two polymer materials contact and separate, they generate an alternating electrical signal. The working principle of the triboelectric nanogenerator (TENG) aligns with Maxwell’s equations of displacement current, which describe this phenomenon as follows [29,30]:

(1) $$J_{D}=\frac{\partial D}{\partial t}=\varepsilon \frac{\partial E}{\partial t}+\frac{\partial P_{S}}{\partial t}$$

In which *D* is the vector field of electric displacement, *ε* represents the dielectric constant, *E* is the electric field, defined as the electric force per unit charge, and *Ps* is the polarization contribution from the triboelectric charging effect on the surface of the dielectric material. No charge is generated in the initial state (separation state I), resulting in no potential difference between the two copper electrodes. As the PVA approaches the polymer (PE, PVDF, PTFE) in state II, a friction-induced charge transfer occurs, creating charges of equal magnitude, but opposite polarity, on the electrodes. The potential difference drives electrons from the lower electrode to the upper electrode. At this point, the top electrode reaches a higher potential, driving the current through the external resistance. During full contact (state III), the surface charges neutralize, stopping the current flow through the external resistance. When the two contact materials separate again, the distance between the layers increases, causing the potential of the lower electrode to be higher than that of the upper electrode. This leads to electrons flowing from the upper electrode to the lower electrode, reducing the amount of induced charge on the electrodes (stage IV). A new working cycle commences and the P-TENG generates a periodic, alternating electrical signal.

We used COMSOL software (version 6.2) to simulate the electric potential distribution during a P-TENG (PVA–PTFE) working cycle. As depicted in Figure 4B, the potential distribution varies with the distance between the PVA and the other polymer, aligning with the described working mechanism of the P-TENG. Lastly, we applied a theoretical model to elucidate the mechanism underlying the CE effect between the PVA and the other polymers. The results, shown in Figure 4C, indicate that when the polymers are separated (state I), their electron clouds do not overlap, and the interfacial potential prevents electron transfer, resulting in no charge transfer. During contact (state II), the overlapping surface electron clouds reduce the interfacial potential barrier, facilitating electron transfer from the PVA to the other polymer. Upon separation (state III), the PVA atoms lose electrons and the polymer atoms gain electrons, resulting in a positively charged PVA surface and a negatively charged polymer surface. In our model, it is crucial to emphasize that electron transfer is the primary theoretical cause of the CE effect in typical conditions.

**Figure 5:**
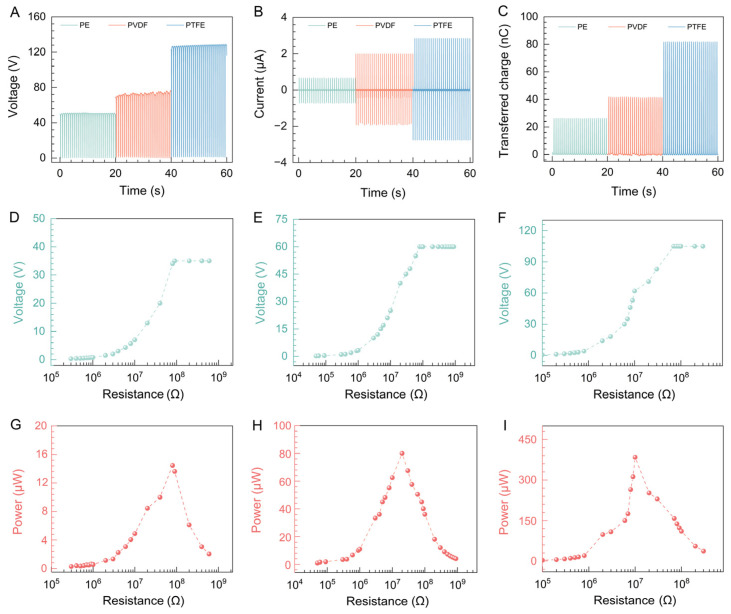
Electric output performance of different polymers in contact–separation with PVA fiber membrane, including voltage (**A**), current (**B**), and transferred charge (**C**). Measurement of the voltage of the P-TENG under different external load resistances at a frequency of 3 Hz and 5 N force, with PVA–PE (**D**), PVA–PVDF (**E**), and PVA–PTFE (**F**). Measurement of the peak power of the P-TENG under different external load resistances at a frequency of 3 Hz and 5 N force, with PVA–PE (**G**), PVA–PVDF (**H**), and PVA–PTFE (**I**).

To assess the output performance of PVA during contact separation with PE, PVDF, and PTFE, we applied pressure using a linear motor and recorded the *V*_OC_, *I*_SC_, and *Q*_SC_. Figure 5A–C shows that under identical conditions, PE had the lowest output (*V*_OC_ = 51 V, *I*_SC_ = 0.37 μA, *Q*_SC_ = 26 nC), PVDF had stronger output (*V*_OC_ = 74 V, *I*_SC_ = 1.98 μA, *Q*_SC_ = 42 nC), and PTFE had the highest output (*V*_OC_ = 128 V, *I*_SC_ = 2.83 μA, *Q*_SC_ = 82 nC). To assess the output when different polymers contact PVA fiber membranes, we measured the output voltage across external load resistances ranging from 10 KΩ to 1 GΩ. The results shown in Figure 5D–F indicate load resistances of 80 MΩ for PE, 20 MΩ for PVDF, and 10 MΩ for PTFE. We used the formula P = *U*^2^/*R* to calculate the output power, where *U* is the output voltage and *R* is the load resistance. As Figure 5G–I shows, the maximum output power was 14.45 μW for PE, 80 μW for PVDF, and 384 μW for PTFE. Based on these findings, PTFE generated the highest output, followed by PVDF, with PE producing the lowest output.

**Figure 6:**
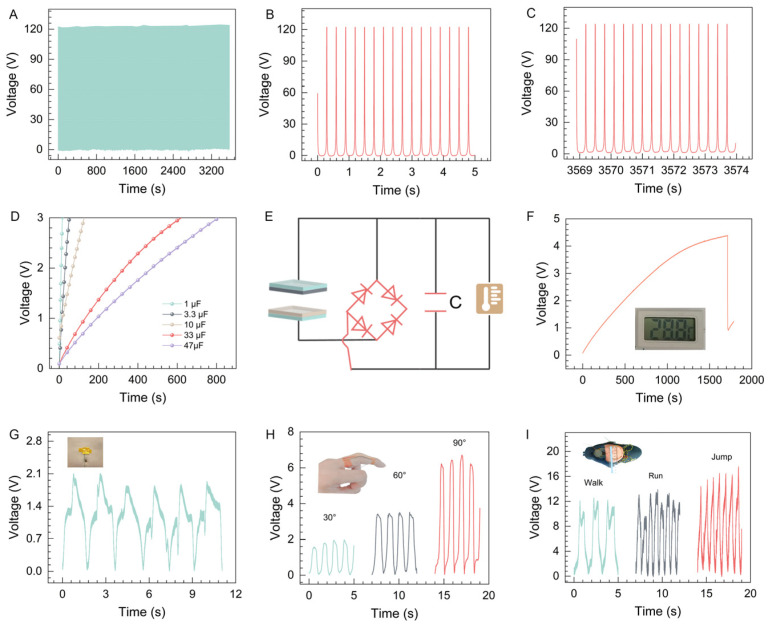
(**A**) Durability test of the P-TENG, first 5 s (**B**), last 5 s (**C**). (**D**) Capacitive charging curve for the P-TENG. (**E**) Working circuit diagram of the TENG supplying power to a thermometer. (**F**) Temperature gauge charging curve of the P-TENG. (**G**) Respiratory monitoring by the P-TENG. (**H**) Finger bending monito by the P-TENG. (**I**) Motion state monitoring by the P-TENG.

The stability of the device is essential for the practical application of PVA in TENG technology. We evaluated the working stability of the P-TENG, using PTFE as a contact material with PVA, as shown in Figure 6A. After 10,000 continuous contact–separation cycles, the *V*_OC_ remained stable. Examination of the initial and final *V*_OC_, as depicted in Figure 6B,C, confirmed *V*_OC_ stability at 122 V, before and after the test. This underscores the exceptional stability and durability of the P-TENG for long-term usage, emphasizing the broad application potential of PVA. To further explore the feasibility of the P-TENG as a self-powered device, we assessed its capacitive charging capabilities. Figure 6D shows the capacitive charging curve of the P-TENG under a force of 5 N, demonstrating its ability to charge a 47 μF capacitor to 3 V in 800 s. We integrated a thermometer with the capacitor to explore powering micro-devices, as shown in Figure 6E. After charging for 28 min, the device activated the thermometer, as demonstrated in Figure 6F. This experiment highlights the potential of PVA-based TENGs to power smart devices. We also validated the potential of PVA in smart sensing technologies by affixing the P-TENG to the abdomen to monitor human respiration, as shown in Figure 6G. The P-TENG displayed electrical signals synchronized with breathing patterns, confirming its effectiveness as a human respiratory sensor. By securing the P-TENG to a finger with tape, we assessed its efficacy as a motion sensor, as depicted in Figure 6H. The electrical signals rose with the bending angle of the finger from 30° to 90° and stayed stable at each angle. To further verify the feasibility of PVA for monitoring vigorous physical activities, we placed the P-TENG in a shoe insole to monitor walking, running, and jumping, as shown in Figure 6I. The electrical signals from the P-TENG peaked during jumping, were lower during running, and were the lowest during walking, with clear peaks for each activity. The excellent stability and sensing performance indicate that PVA materials are well-suited for self-powered and sensor applications in smart devices.

## 4. Conclusions

This study elucidates the CE mechanism between PVA and other polymers (PE, PVDF, PTFE) and identifies its potential for practical applications in the field of TENGs. By analyzing the energy gap between the HOMO and the LUMO, it can be demonstrated that PVA is more susceptible to electron transfer. It is worth noting that when PVA and PTFE molecules come into contact, the charge transfer primarily occurs within the outermost electron layer of the molecules. In the course of the performance experiments, the highest output performance was also achieved with a *V*_OC_ of 128 V, an *I*_SC_ of 2.83 µA, a *Q*_SC_ of 82 nC, and a power output of 384 µW. The P-TENG was prepared by combining PVA and PTFE, and its capacity to power a thermometer was successfully verified. Furthermore, the P-TENG demonstrated the capacity to accurately monitor human respiratory activity, finger flexion, and movement status. This study not only advances our comprehension of the mechanism of action of PVA in the domain of friction nanogenerators, but also furnishes insights that can inform prospective innovations in self-powered and wearable electronic devices.

## Data Availability

The data are contained within the article.
